# Mechanical and Durability Performance Improvement of Natural Hydraulic Lime-Based Mortars by Lithium Silicate Solution

**DOI:** 10.3390/ma13225292

**Published:** 2020-11-23

**Authors:** Zijian Song, Zhongyuan Lu, Zhenyu Lai

**Affiliations:** 1 State Key Laboratory for Environment-Friendly Energy Materials, School of Materials Science and Engineering, Southwest University of Science and Technology, Mianyang 621010, China; laizhenyu@swust.edu.cn; 2 Department of Material Engineering, Mianyang Vocational and Technical College, Mianyang 621000, China

**Keywords:** lithium silicate, natural hydraulic lime, pores structure, compressive strength, durability

## Abstract

Natural hydraulic lime (NHL) as a building material has been widely used to restore the historic structure. However, the slow growth rate of strength and durability limits its engineering application. In this work, the NHL-based mortars were pretreated by lithium silicate (LS) solution impregnation and surface spraying. The results show that the compressive strength, surface hardness, and freeze-thaw cycle (FTC) resistance of NHL-based mortar were greatly improved after LS pretreatment. Specifically, the compressive strengths of the sample increased by about 32.7–52.0%. LS was sprayed on the sample’s surface (about 0.2 kg/m^2^) and the surface hardness increased by up to 10 grades. Compared with the control samples, the weight loss of treated samples decreased by about 31.6–43.8%. A rehydration process to generate the hydrated calcium silicate gel (C-S-H) was observed between calcium hydroxide (CH) and LS, which can decrease the sample’s porosity and form a silicate coating on the surface. With an increase in the concentration of LS, the macropores (50–10,000 nm) content decreased, while the mesopores (10–50 nm) and nanopores (less than 10 nm) increased. This work reveals that the LS pretreatment provides a potential route to improve NHL-based mortar’s mechanical properties and durability.

## 1. Introduction

Natural hydraulic lime (NHL) is an important inorganic binder that can be hydrated in air and water [1,2,3]. Three classes of NHL were defined by the new version of the European Standard (EN459-1:2015) based on the compressive strength after 28 days of curing and the content of calcium hydroxide (CH): NHL2, NHL3.5, and NHL5 [4]. In NHL, C_2_S is the major hydraulic phase, while the calcium hydroxide (CH) is contributed to the carbonation for hardening [5,6,7]. Due to NHL-based mortars having low shrinkage [8], good permeability, and compatibility with old materials, NHL is widely used to repair historical structures and the preparation of fluid screeds [9,10]. However, certain disadvantages of NHL, such as the slow growth rate of strength and low final strength, limit its wide application.

Multiple additives have been used to improve the mechanical performance and its durability. For example, pozzolan’s introduction as a partial substitution of NHL has been studied [11,12,13]. Grilo et al. [14] reported that the mechanical strength of NHL-metakaolin (NHL-MK) mortar increased with the introduction of metakaolin to replace lime partially. However, with an increase in aging time, the NHL-MK mortar’s compressive strength would decrease due to the increase of kaolin content. A similar result is reported by Luso et al. [15,16] in which the compressive strength of the injection grout with a ternary binder experienced a decrease after 180 days of aging. The poor durability is ascribed to the instability of calcium-aluminum hydrated compounds formed in the pozzolanic reaction [17]. In addition, Luis G. et al. [18] have investigated the effect of silica fume (SF) on the properties of NHL-based grouts. The mechanical strength was increased with an increase of SF dosages. However, the workability decreased gradually. Furthermore, the mechanical and physical properties have been improved by adding graphene oxide (GO) in NHL-based mortar [19]. The above studies mainly focused on the modification of lime as a restoration material for historic buildings. However, the issues of the slow growth of strength and surface hardness remain, which are of critical importance to explore the wide scope of application of NHL in engineering.

Lithium silicate (Li_2_O·nSiO_2_) (LS) is one of the typical lithium salt, where n is the modulus of LS and is defined by the mole ratio of SiO_2_/Li_2_O. Since the lithium ion’s radius is only 0.0768 nm, LS solutions with a larger modulus can be prepared without affecting the viscosity of the solution. LS is widely used as an anti-corrosive coating for the surface of steel bars and the surface treatment of cement-based materials [20]. Witzleben [21] has systematically investigated the effects of the addition of LS in cement, including the setting time, strength, heat of hydration, and phase formation of cementitious systems. Abou Sleiman et al. [22] reported that the LS could form insoluble wear and moisture-protective surface on the concrete’s surface. Stepien et al. [23] used LS to modify silicate autoclaved material in which the improved compressive strength was obtained. The corresponding chemical reaction between LS and CH Equation (1) is:

Li_2_O·nSiO_2_ + mH_2_O + nCa(OH)_2_→nCaO·SiO_2_·(m + n − 1)H_2_O + 2LiOH (1)

It shows that the existence of LS leads to part of CH forming new calcium silicate gel (C-S-H), which gives rise to the improved densification of the hydration products. Previous studies have shown that there is a large amount of CH in NHL, but the carbonation is a long-term process [24,25]. The existence of a large amount of CH in NHL-based mortar would adversely affect its strength, surface hardness, and durability [26,27]. Hence, converting the CH to C-S-H could dramatically improve the serviceability of NHL.

In this paper, we focus on the effects of the LS modification on NHL-based mortars’ mechanical and durability. To verify whether LS could improve NHL-based mortar properties, the compressive strength, surface hardness, air permeability, water absorption, and FTC resistance were tested after pretreatment. The micro-morphology, pore structures, and thermogravimetric (TG) analysis were used to investigate the mechanism of the effect of LS on NHL-based mortar.

## 2. Materials and Methods

### 2.1. Materials

**Figure 1:**
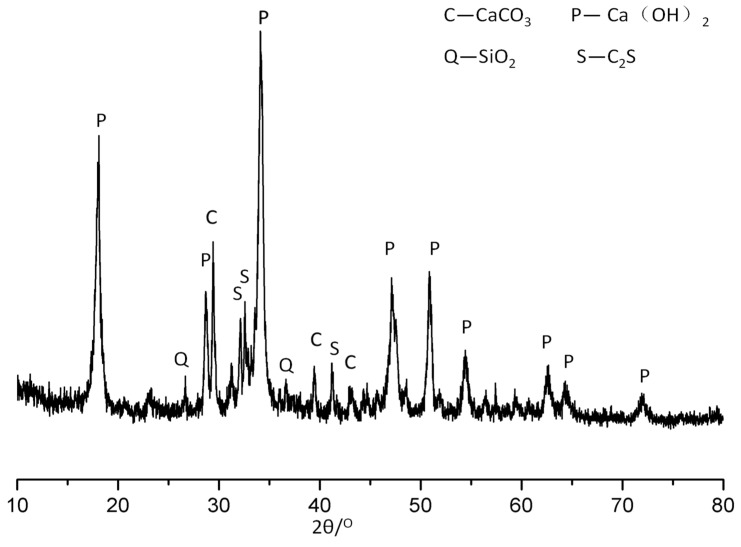
X-ray diffraction pattern of NHL2.

In this study, NHL2 was purchased from Hessler Kalkwerke GmbH (Hessler, Germany) following the European standard EN459-1:2015. Chemical composition and X-ray diffraction patterns of NHL2 are shown in Table 1 and Figure 1, respectively. The LS was supplied by Specchem LLC (Shanghai, China), diluted with deionized water into three different concentrations. The concentration was 5.0%, 10.0%, and 15.0%, respectively. The aggregates are quartz sand with particle diameters ranging from 0.5 to 2.0 mm. Tap water was used in the preparation of the NHL-based mortar samples.

### 2.2. Preparation

In this investigation, the mass ratio of water/NHL2 is 0.5, and sand/NHL2 is 1:1, 2:1, and 3:1, respectively. The mix proportion and pretreated details are listed in Table 2. According to the mixing ratio, the raw materials were mixed in the mortar mixer. The metallic mould is 40 mm × 40 mm × 40 mm for the compressive strength, water absorption, and FTC tests (Three samples are needed for each group, and average values are calculated). The metallic mould is 20 mm × 100 mm × 100 mm for the hardness and permeability tests (Three samples are needed for each group, and average values are calculated). After molding, all the samples were cured for 24 h (RH = 100% and T = 21 ± 1 °C), then demolded and cured (RH = 50% and T = 21 ± 1 °C). After curing for 3-days, 7-days, and 28-days, the samples for compressive strength measurement were dried to a constant weight (T = 50 °C) and then submerged in LS solution for 8 h. After soaking for 8 h, all samples were taken out and dried to a constant weight in the oven (T = 50 °C). The samples for air permeability index (API) and hardness tests are cured for 7-days and 28-days (RH = 50% and T = 21 ± 1 °C) and then dried to constant weight before the LS solution was sprayed onto the surface (about 0.2 kg/m^2^). Similarly, the samples for FTC and water absorption tests are cured for 28-days (RH = 50% and T = 21 ± 1 °C) and then dried to a constant weight before the LS solution was sprayed onto the surface (about 0.2 kg/m^2^). When the compressive strength test was completed, samples were broken and screened as required for SEM, TG test (Remove sands from samples), and MIP tests (The particle diameter is about 5 mm).

### 2.3. Test Methods

**Figure 2:**
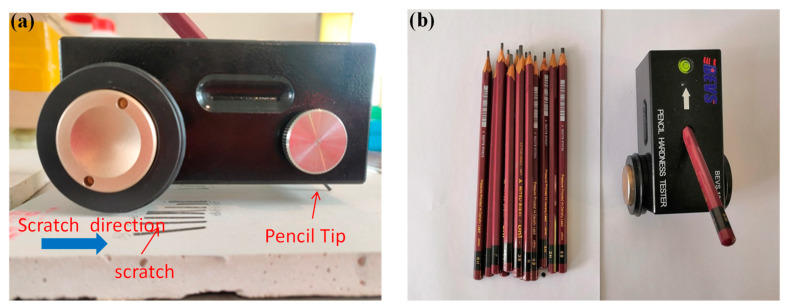
Pencil hardness tester. (**a**) Pencil hardness tester. (**b**) Pencil.

The pencil hardness tester (Figure 2) (3BEVS1301, Huasheng, Guangzhou, China) was used for the hardness test in accordance with the ASTM D3363-2005 [28]. This test method covers a procedure for the rapid, inexpensive determination of the substrate’s surface hardness in drawing leads or pencil leads of known hardness. There are 14 grades of pencil hardness tester, from low to high, 6B, 5B, 4B, 3B, 2B, B, HB, F, H, 2H, 3H, 4H, 5H, and 6H, respectively. The weight of the complete hardness tester was about 19.6 N and the weight transferred by the tip of the pen is about 6.9 ± 5 N. When testing, the sample was placed horizontally, and the hardness tester was placed on the surface of the sample. The pencil formed a 45° angle with the tested surface. The pencil hardness tester was pushed forward horizontally at a speed of 1 cm/s for 2 cm.

The NHL mortars’ compressive strengths were evaluated on the testing machine (SANS CMT5105, Shenzhen, China) at a loading rate of 2400 ± 200 N/s. The treated samples and the control samples were cured for 3-days, 7-days, and 28-days under the same conditions. The compressive strength values at different stages were obtained to evaluate LS’s effect on the NHL-based mortar strength.

**Figure 3:**
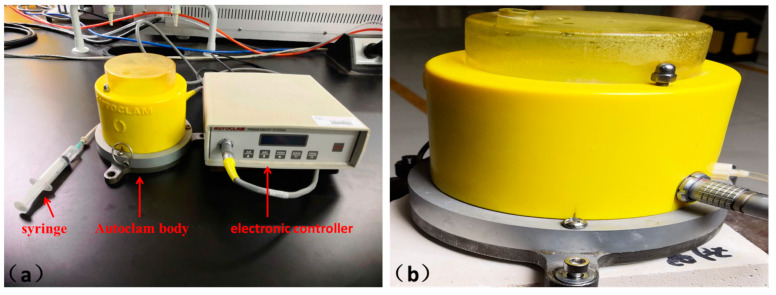
Autoclam permeability system. (**a**) Syringe for applying air pressure, autoclam body an delectronic controller. (**b**) The sample being tested

The Autoclam permeability system (Figure 3) was developed by Queen’s University Belfast to measure the sorptivity, air permeability, and water permeability of concrete [29,30]. This system was adopted to test the air permeability of the NHL-based mortar. During the test, the air was injected into the Autoclam body through a syringe. When the air pressure reached 0.5 bars, the electronic controller started to work, and a pressure decrease was monitored every minute until the pressure diminished to zero. A plot of the natural logarithm of air pressure against time is linear, and the slope of the linear regression curve for tests is used as an air permeability index (API). The logarithmic function is calculated by Equation (2).

AI = ln P/t(2)

where AI is the logarithmic function value, P is the air pressure, and t is the pressure corresponding to time.

To evaluate the effect of LS on FTC resistance of NHL-based mortar, the weight loss was investigated after FTC. After 28-days of curing, the NHL-based mortar was initially immersed in tap water until obtaining a saturated state. An FTC includes the following processes. First, the samples were set in a −15 °C freezer for 4 h. Then this was followed with a thawing period, in which the samples were immersed in water at room temperature for 4 h. The weight loss of samples was calculated by Equation (3).

(3) $$W\;=\;\frac{W_{0}-W_{n}}{W_{0}}\times 100$$

where W is the weight loss of FTC (%) for n times, *W*_0_ is the initial weight, and *Wn* is the weight of samples after FTC for n times.

The surface morphology of the samples was characterized by SEM (UItra55, Carl Zeiss NTS GmbH, Heidenheim, Germany). The matrix’s pore structure was represented by MIP (Auto pore IV9500, Micromeritics, Norcross, GA, USA, maximum mercury intrusion pressure: 30,000 Psi). Thermogravimetry (TG, Jupiter STA449C, Netzsch, Bavaria, Germany) was carried out at 10 °C/min in an atmosphere of flowing N_2_ (50 mL/min) in alumina crucibles over a temperature ranging from 25 to 1000 °C.

## 3. Results and Discussion

### 3.1. The Effect on Mechanical Properties

**Figure 4:**
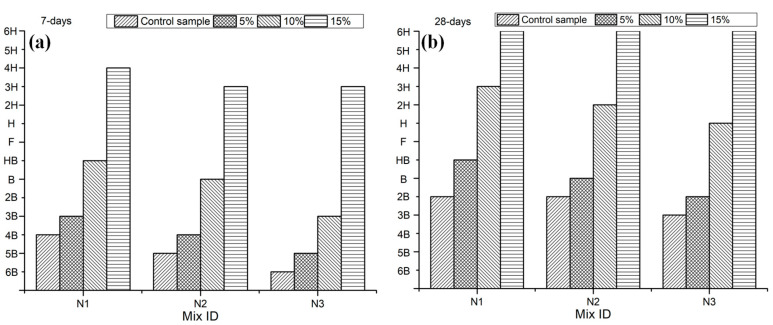
Effect of LS on the hardness of NHL-based mortar. (**a**) Modification after 7-days of curing. (**b**) Modification after 28-days of curing.

NHL-based mortars were treated with three different concentrations of LS. The effect of the LS pretreatment on the hardness of NHL-based mortar is shown in Figure 4. In Figure 4, samples’ surface hardness at 7-days and 28-days increased significantly when LS solution concentration gradually increases from 5.0% to 15.0%. Specifically, after treatment by LS, the 7-days surface hardness grades of sample N1-3, N2-3, and N3-3 reach 4H, 3H, and 3H, respectively. Similarly, the surface hardness of the sample cured for 28-days can also significantly increase with the LS surface treatment. The 28-days surface hardness grades of sample N1-3, N2-3, and N3-3 reach 6H. This phenomenon can be attributed to the fact that a large amount of CH in NHL2 is consumed by LS to form C-S-H, forming a silicate coating on the sample’s surface [6,21]. The sand/NHL2 ratio has an important effect on NHL-based mortar’s surface hardness. It can be concluded that a lower sand/NHL2 ratio is more likely to improve the surface hardness. It is similar to the results reported by Pan et al. [31]. The magnesium fluorosilicate and sodium silicate decreased the carbonation depth and increased surface hardness of concrete due to the formation of silicate coating by C-S-H. In addition, Thompson et al. [32] reported that sodium silicate could improve concrete surface properties such as hardness, permeability, chemical durability, and abrasion resistance.

**Figure 5:**
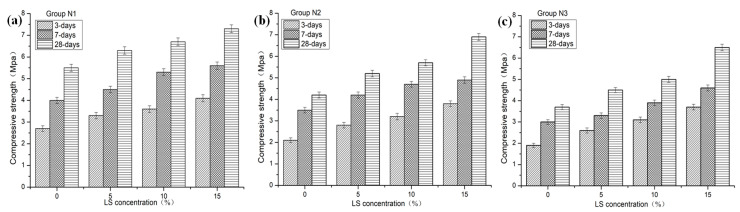
Effect of LS on compressive strength of NHL-based mortar: (**a**) Group N1, (**b**) group N2, and (**c**) group N3.

As shown in Figure 5, the compressive strength test results revealed that the compressive strengths of samples treated by LS were higher than that of the control samples. The compressive strength increases with an increase in LS concentration. Specifically, the compressive strengths of the sample N1-3 were increased by 52.0% (3-days), 40.0% (7-days), and 32.7% (28-days), respectively. Similarly, the compressive strengths of the sample N2-3 were increased by 81.0% (3-days), 40.0% (7-days), and 64.3% (28-days). The compressive strength of the sample N3-3 were increased by 94.7% (3-days), 53.3% (7-days), and 75.7% (28-days), respectively. The increased compressive strengths were attributed to the introduction of more silicate ions by the higher concentration of LS solution, which consumed CH to form more C-S-H. This result agrees well with the results reported by Luis G [18] in which the silica fume (SF) leads to pozzolanic reactions by reaction with CH, resulting in the formation of additional C-S-H.

### 3.2. The Effect on Air Permeability

**Figure 6:**
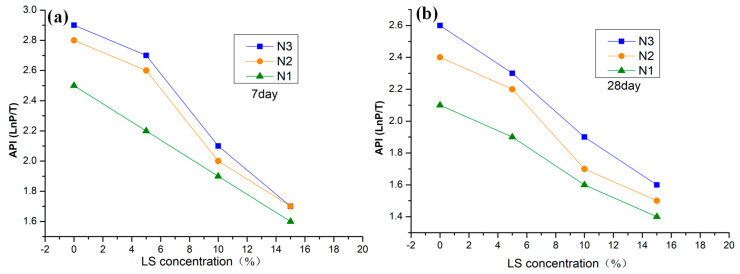
Effect of LS on API of NHL-based mortar. (**a**) Modification after 7-days of curing. (**b**) Modification after 28-days of curing.

Autoclam air permeability testing was adopted to investigate the effects of LS pretreatment on the air permeability of NHL-based mortar [33]. As shown in Figure 6, the API value of samples showed a decreasing trend when LS’s concentration gradually increased from 0% to 15.0%. Specifically, the API value of N1-3, N2-3, and N3-3 at 7-days were 1.7, 1.7, and 1.6, respectively. A dramatic decrease occurred at 28 days when the API value of N1-3, N2-3, and N3-3 were 1.6, 1.5, and 1.4 after being pretreated with 15.0% LS solutions. It can be ascribed to the formation of a large amount of C-S-H within 8 h. C-S-H formed silicate coating on the samples’ surface that blocks the capillary pores, which could reduce the erosion of harmful substances and improve NHL-based mortar durability [34]. The result was consistent with previous results reported by J. LaRosa Thompson, which demonstrated that silicate sealers could decrease the air permeability [32].

### 3.3. Effect of LS on Water Absorption of NHL-Based Mortar

After curing for 28-days, the water absorption of the sample was tested after being treated by LS [30]. The samples were first vacuumed for 4 h, and then immersed in deionized water for 48 h. After wiping the surface water, the mass (*m*_1_) of the samples were measured under a surface-dry condition, and then the samples were dried in an oven at 80 °C until reaching constant mass (*m*_0_). The water absorption of the samples can be calculated using Equation (4).

(4) $$\rho =\frac{m_{1}-m_{0}}{m_{\begin{array}{c} 1 \end{array}}}\times 100$$

**Figure 7:**
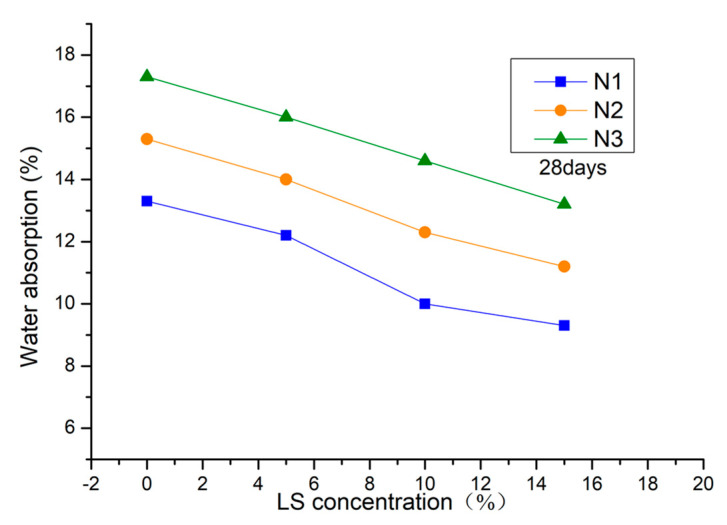
Effect of LS on water absorption of NHL-based mortar.

The effect of LS pretreatment on water absorption of NHL-based mortar is shown in Figure 7. As shown in Figure 7, the water absorption of mortar decreases gradually after pretreatment. This result agrees well with the API testing. When compared with the control samples, the water absorptions of the group N1, N2, and N3 decreased by 43.0%, 36.6%, and 31.6% at 28-days after 15.0% LS solution pretreatment. Water absorption reflects the porosity of concrete. Thus, it represents that the LS solution reduced permeability performance by decreasing the porosity of NHL-based mortar. It has been reported that inorganic and organic materials are used to prepare coatings of cement-based materials. Relevant literature [32] reveals that the sodium silicates (SS) can prevent erosion with 15% concentration of chloride ion and that the resistance of freezing-thawing of coated samples is improved due to the reduced water absorption [35]. Although organic surface treatment agents can significantly reduce water absorption, they may lose their protective effect under high temperature and weathering [36].

### 3.4. The Analysis of Morphology

**Figure 8:**
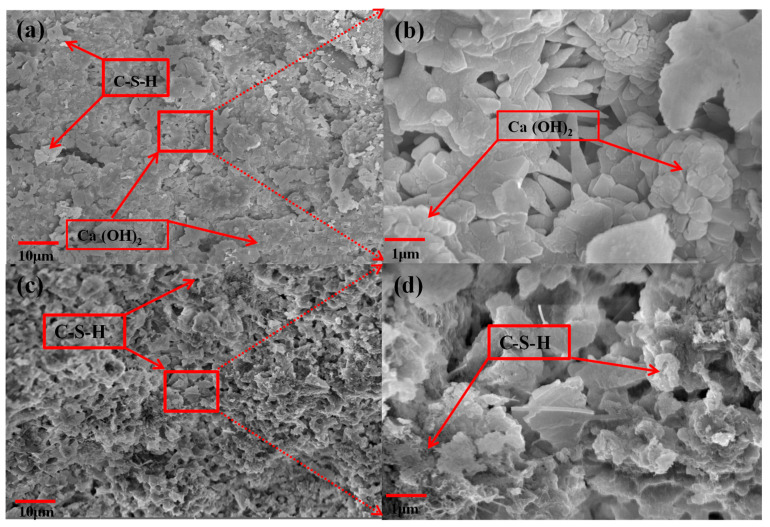
SEM images of (**a**,**b**) samples N3-0, (**c**,**d**) samples N3-3.

The micro-morphology of the samples N3-0 cured for 3 days and sample N3-3 treated with LS after being cured for 3 days are shown in Figure 8. As shown in Figure 8a,b, a large number of CH and a few C-S-H are observed. These products are mainly from the raw materials and partly from the hydration products of C_2_S. The edges of CH grains are clear and overlapped, which leads to larger micro-pores, microvoids, and micro-cracks in sample N3-0.

In Figure 8c,d, CH almost disappeared, and a large number of amorphous phases were observed. It can be observed that a dense C-S-H coating has been formed on the surface of sample N3-3. The high concentration of silicate ions indicated that a large amount of CH was consumed by LS to form C-S-H. These results are consistent with the permeability test. The pores are gradually filled, giving rise to a decrease in air permeability when more C-S-H gel is formed [30,32].

### 3.5. Effect of LS Pretreatment on FTC Resistance

**Figure 9:**
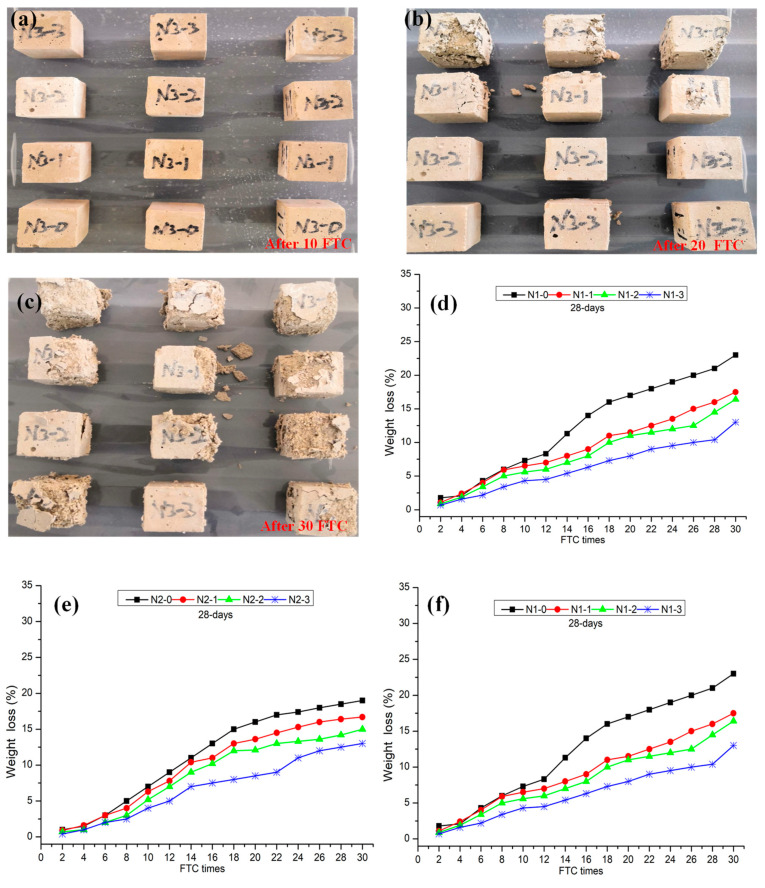
Photographs of the group N3 after FTC tests: (**a**) After 10 FTC, (**b**) After 20 FTC, (**c**) After 30 FTC. Weight loss with FTC for NHL-based mortar: (**d**) Group N1, (**e**) group N2, and (**f**) group N3.

Figure 9a–c show the damage of group N3 after 10, 20, and 30 FTC tests, respectively. As shown in Figure 9a, the surface separation occurred on the surface of sample N3-0 after 10 FTC, and the sample N3-1, N3-2, and N3-3 still maintained an intact state. In comparison, the control samples occurred with a cataclastic failure after 20 and 30 FTC, respectively. It is verified by the treated samples to have larger FTC resistance when compared to the control samples. Particularly, the higher concentration of LS was more beneficial to improve the FTC resistance. Similarly, group N1 and group N2 show the same trend after FTC tests. Figure 9d–f shows the weight loss of group N1, N2, and N3 after 30 FTC. Compared with the pretreated samples, the weight loss of N1-0, N2-0, and N3-0 after 30 FTC were 23.0%, 19.0%, and 32.0%, respectively, while the weight loss of sample N1-3, N2-3, and N3-3 after 30 FTC were 13.0%, 13.0%, and 18.0%, respectively. These results are very different from previous reports. Shuqiang Xu et al. [37] reported that the compressive strength is much higher after the FTC tests, which could be attributed to the water curing condition that can further improve the mortars’ strength.

### 3.6. Pore Structures

**Figure 10:**
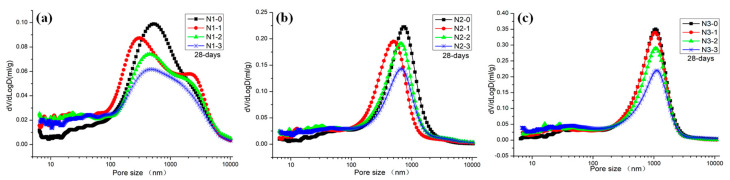
Effect of LS pretreatment on the pore size distribution of NHL mortar: (**a**) Group N1, (**b**) group N2, and (**c**) group N3.

**Figure 11:**
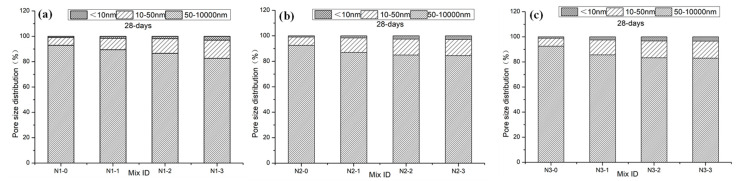
Percent of macropores, mesopores, and nanopores of the samples after modification: (**a**) Group N1, (**b**) group N2, and (**c**) group N3.

Figure 10 presents the effect of LS modification on the pore structure distribution of NHL-based mortar. A main peak was located at 1000 nm in the pore size distribution curves, which is the same as the previous studies [5]. When the concentration of LS increased from 5% to 15.0%, the peaks around 1000 nm decreased gradually. As shown in Figure 10b,c, these peaks moved to the position of 700 nm and 400 nm, respectively. It indicates that the void ratio of macropores (50–10,000 nm) in the samples decreases gradually with an increase in the concentration of LS. In contrast, the void ratio of nanopores (less than 10 nm) increased gradually. Porosity and pore size distribution through MIP data are shown in Table 3 and Figure 11, respectively.

The porosity of samples with a different void ratio show a decreasing trend with an increase in LS concentration. In the meantime, the number of macropores (50–10,000 nm) decreased while mesopores (10–50 nm) and nanopores (less than 10 nm) increased with an increase in LS concentration. The void ratio of macropores in the sample decreases gradually. Compared with the control samples, macropores of the samples N1-3, N2-3, and N3-3 gradually decreased from 92.9% to 82.7%, 92.5% to 84.4%, and 92.6% to 83.0%, respectively. On the contrary, with LS concentration increasing, the void ratio of mesopores and nanopores in the sample gradually increases. The mesopores of the samples N1-3, N2-3, and N3-3 gradually increased from 6.1% to 14.3%, 6.5% to 12.5%, and 6.3% to 13.6%, respectively. Mesopores of the samples N1-3, N2-3, and N3-3 gradually increased from 6.1% to 14.3%, 6.5% to 12.5%, and 6.3% to 13.6%, respectively. Nanopores of the samples N1-3, N2-3, and N3-3 gradually increased from 1.0% to 3.0%, 1.0% to 2.9%, and 1.1% to 3.4%, respectively. The increase of mesopores and nanopores with the addition of LS mainly attributed to the filling effect of C-S-H formation through rehydration of CH and LS [21]. It is similar to the results reported by Kai Luo [38,39].

### 3.7. TG Analysis Results

**Figure 12:**
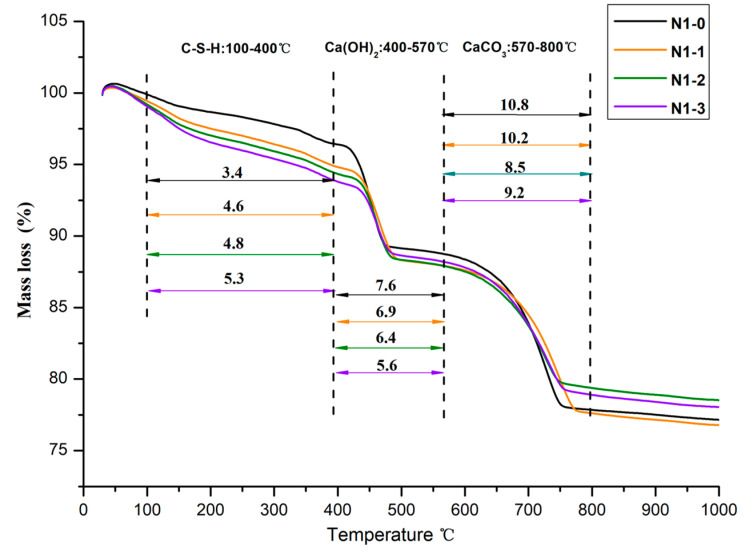
TG curves of the group N1.

Figure 12 shows the thermogravimetric (TG) curve of group N1 cured for 28-days with and without LS pretreatment. Four mass loss stages could be observed, including 0–100 °C, 100–400 °C, 400–570 °C, and 570–800 °C, respectively. The mass loss stage below 100 °C is corresponding to the dehydration of adsorption water. The mass loss at 100–400 °C corresponds to dehydration of C-S-H, while the mass loss at 400–570 °C is the dehydroxilation of CH. The mass loss at 570–800 °C is due to the decomposition of calcium carbonate (CC) [40]. Based on each mineral’s mass loss characteristics at an elevated temperature, the content of water bound to C-S-H (%), CH, and CC in hardened NHL pastes could be evaluated [41,42]. As shown in Figure 12, with an increase in LS concentration, the content of C-S-H increased, while the content of CH and CC decreased. The detailed content of C-S-H, CH, and CC in hardened pastes after being treated by LS are given in Table 4.

It can be observed that the addition of LS can regulate the content of C-S-H. Compared with the control sample, the C-S-H content of the group N1, group N2, and group N3 increased by 55.9%, 48.6%, and 60.6%, respectively. On the contrary, the addition of LS can decrease the content of CH and CC. In particular, CH’s content decreased by 35.9%, 36.1%, and 34.0% when the concentration of LS was 15.0%, respectively. At the same time, the CC content decreased by 16.7%, 36.7%, and 31.9%, respectively. The results show that LS could consume exising or newly generated CH to form C-S-H. These results agree well with the results of pore structures analysis and the air permeability test.

## 4. Conclusions

In this study, NHL-based mortar was pretreated by LS. From the test and analysis results, the following conclusions can be drawn:

1. The NHL-based mortar’s compressive strength can be improved after being impregnated in LS solution for 8 h. The growth rate of compressive strength was maintained between 32.7% and 52.0%.
2. After spraying LS on the sample’s surface (about 0.2 kg/m^2^), the surface hardness increased by up to 10 grades.
3. The sample surface was densified by LS, and the FTC resistance was improved. In particular, compared with the control samples after 30 FTC tests, the weight loss of sample N1-3, N2-3, and N3-3 decreased by 43.0%, 31.6%, and 43.8%, respectively.
4. LS can consume existing or newly generated CH to form C-S-H, which refines the NHL-based mortar’s pore structure, leading to a decrease in API value and water absorption.

## Figures and Tables

**Table 1:** Chemical composition of NHL2 (wt %).

Chemical Composition	SiO_2_	CaO	Fe_2_O_3_	MgO	Al_2_O_3_
NHL2	9.48	78.66	1.98	4.51	3.12

**Table 2:** The mix design of NHL-based mortar.

Samples	Composition of Mixture (kg/m^3^)					LS Concentration (%)
	Sand/NHL2	Water/NHL2	NHL2	Sand	Water	
N1-0	1:1	0.5	450	450	225	0
N1-1						5
N1-2						10
N1-3						15
N2-0	2:1	0.5	450	900	225	0
N2-1						5
N2-2						10
N2-3						15
N3-0	3:1	0.5	450	1350	225	0
N3-1						5
N3-2						10
N3-3						15

**Table 3:** Effect of LS on porosity of NHL-based mortar cured for 28-days (wt %).

Concentration of LS	Group N1	Group N2	Group N3
0%	25.58	29.84	37.71
5.0%	23.58	29.20	36.72
10.0%	23.11	28.38	34.72
15.0%	20.12	26.38	31.83

**Table 4:** The content of the main phases in samples with various concentrations of LS (%).

Group	Samples	Water Bound to C-S-H	CH	CC
N1	N1-0	3.4	31.4	24.5
	N1-1	4.6	28.3	23.3
	N1-2	4.8	26.5	19.2
	N1-3	5.3	23.1	21.0
N2	N2-0	3.5	32.4	24.6
	N2-1	4.3	29.2	23.7
	N2-2	4.5	27.3	19.8
	N2-3	5.2	23.8	18.0
N3	N2-0	3.3	32.3	24.0
	N2-1	4.7	29.3	23.0
	N2-2	4.6	27.4	19.1
	N2-3	5.3	24.1	18.2
